# The Effect of the DASH Diet on the Development of Gestational Hypertension in Pregnant Women: A Systematic Review and Meta-Analysis

**DOI:** 10.3390/nu18020268

**Published:** 2026-01-14

**Authors:** Anastasios Alatsis, Nikoleta Aikaterini Xixi, Rozeta Sokou, Paraskevi Volaki, Styliani Paliatsiou, Zoi Iliodromiti, Nicoletta Iacovidou, Theodora Boutsikou

**Affiliations:** 1 School of Medicine, National and Kapodistrian University of Athens, 11528 Athens, Greece; 2 Neonatal Department, Aretaieio Hospital, School of Medicine, National and Kapodistrian University of Athens, 11528 Athens, Greecerosesok@med.uoa.gr (R.S.);

**Keywords:** pregnancy preeclampsia, diet, hypertensive disorders of pregnancy

## Abstract

**Background/Objectives**: Gestational hypertension is associated with increased maternal and fetal morbidity. The DASH diet is designed to reduce blood pressure and improve cardiovascular health. Our aim is to evaluate the efficacy of adherence to the DASH dietary pattern during pregnancy on the incidence of GH. **Methods**: PubMed, Scopus, Web of Science, Cochrane Library and Embase were systematically searched. All studies including data on the effect of the DASH diet on GH were included in this review. The study is registered in PROSPERO (CRD420251044348). **Results**: A total of five studies were included in our study. The meta-analysis reported a pooled relative risk (RR) of 1.03 (CI: 0.86–1.23) for the effect of the DASH diet on gestational hypertension. In the subgroup analysis for preeclampsia, the overall relative risk estimate was 0.78 (95% CI: 0.60–1.02). Both analyses did not yield statistical significance. **Conclusions**: Current evidence, although showing a favorable trend, does not conclude that the DASH diet reduces the risk of gestational hypertension, as the results did not achieve statistical significance. Although potential benefits have been observed, the limited number of available studies does not allow for definitive conclusions. More randomized and multicenter studies are needed to thoroughly investigate the relationship between the DASH diet and gestational hypertension in order to implement this dietary program instead of general dietary recommendations for GH.

## 1. Introduction

Gestational hypertension, defined as blood pressure ≥140/90 mmHg occurring after the 20th week of gestation in previously normotensive women, is a multidimensional entity, with its pathogenesis lying in various pathways [1]. In the proposed pathophysiological mechanisms of gestational hypertension, reduced nitric oxide bioavailability, heightened expression of vasoconstrictors such as endothelin-1, dysregulation of the VEGF/sFlt-1 axis, increased endothelial permeability, and immune maladaptation play a crucial role [2,3]. Established risk factors for developing gestational hypertension include a previous history of the disorder, obesity, nulliparity, maternal age over 35 years, pre-existing diabetes mellitus, multiple gestations, and genetic factors [4].

Gestational hypertension is associated with increased maternal and fetal morbidity, elevating the risk of preterm delivery, either spontaneous or medically indicated, due to complications such as pre-eclampsia (PE), HELLP syndrome, renal and hepatic dysfunction, pulmonary edema, and fetal distress [1,5]. Additionally, gestational hypertension correlates with intrauterine growth restriction (IUGR) and placental abruption, increasing maternal and neonatal morbidity risks [5].

The influence of dietary supplements and macronutrients on the risk of hypertensive disorders of pregnancy is significant. Folate intake, especially early in pregnancy, is associated with reduced risk, although excessive doses may increase risk, underscoring the need for cautious dosing [6,7]. Iron exhibits a complex relationship with hypertensive disorders, whereas calcium demonstrates a clear protective effect, particularly in populations with low dietary intake [7]. Conversely, data on vitamin D remains inconclusive [8]. Consumption of animal-based proteins appears not to directly affect the risk of gestational hypertension, while the roles of fats and carbohydrates remain ambiguous, highlighting the necessity for individualized and balanced nutritional interventions [9,10,11].

Overall dietary patterns before pregnancy may influence the risk of hypertensive disorders of pregnancy. For example, it has been observed that a Mediterranean-style dietary pattern, characterized by vegetables, legumes, nuts, tofu, rice, pasta, rye bread, red wine, and fish, was independently associated with a lower risk of developing hypertensive disorders during pregnancy. In contrast, other dietary patterns, including meat, high-fat and -sugar, fruit and low-fat dairy, and cooked vegetables, were not significantly associated with hypertensive disorder risk. Key contributors to the protective effect of the Mediterranean-style diet included nuts, red wine, and rye bread, suggesting that a combination of foods rich in fiber, unsaturated fats, and micronutrients may be more influential than single nutrients alone [12].

The DASH diet is a scientifically validated nutritional intervention aimed at reducing blood pressure and promoting cardiovascular health through increased consumption of fruits, vegetables, whole grains, lean proteins, and low-fat dairy products, combined with sodium, saturated fat, and added sugar restriction. Its implementation has demonstrated significant improvements in blood pressure, lipid profile, and cardiovascular risk reduction [13,14]. High adherence to this diet correlates with reduced cardiovascular risk and enhanced vascular function, attributable to its nutrient-rich and antioxidant composition, and evidence from controlled trials shows that the diet can progressively reduce biomarkers of subclinical cardiac injury and systemic inflammation over several weeks, indicating benefits beyond traditional cardiovascular risk factors [15,16].

A review involving six randomized controlled trials and 569 participants found that adherence to the DASH eating pattern during pregnancy significantly decreased maternal fasting plasma glucose levels and the incidence of PE. Favorable neonatal outcomes were also noted, specifically reduced incidence of fetal macrosomia and infants who were large for gestational age (LGA) [17].

The established benefits of the DASH diet in lowering blood pressure in the general population suggest that investigation of its role in managing or preventing GH might benefit this category of patients. Since the core mechanisms of the DASH diet, namely improving blood pressure regulation, enhancing endothelial function, and reducing inflammation via its rich content of potassium, magnesium, calcium, antioxidants, and fiber, directly address the pathophysiological hallmarks of hypertensive disorders, its application in pregnancy is promising. Therefore, the objective of this study is to evaluate the efficacy of adherence to the DASH dietary pattern during pregnancy on the incidence of GH.

## 2. Materials and Methods

### 2.1. Protocol and Registration

This systematic review and meta-analysis was performed and reported according to the Preferred Reporting Items for Systematic Reviews and Meta-Analyses (PRISMA) statement [18]. The protocol was registered in PROSPERO (CRD420251044348) and is available online.

### 2.2. Literature Search Strategy

A literature search was conducted across electronic databases to identify relevant studies addressing gestational hypertension and the DASH diet. PubMed, Scopus, Web of Science, Cochrane Library, and Embase were systematically searched from 1 November 2024 until 30 May 2025. Additionally, reference lists of included articles were examined to locate further pertinent studies. Search terms were constructed using combinations of keywords and Boolean operators (AND, OR). The search query employed keywords such as “Gestational Hypertension”, “Pregnancy-induced Hypertension”, “Hypertensive Disorders of Pregnancy”, “DASH diet”, and “Dietary Approaches to Stop Hypertension”. There were no time, language and geographical restrictions.

### 2.3. Study Selection

**Figure 1:**
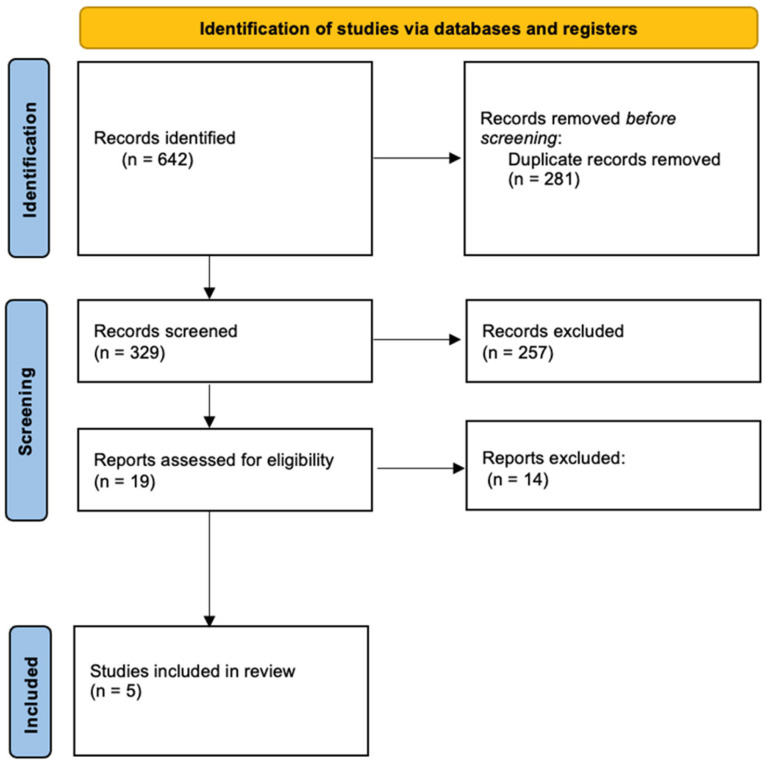
PRISMA flow diagram.

Following removal of duplicates, titles, abstracts, and full texts were screened to identify relevant articles. Studies were included in the meta-analysis if they met the following criteria: (1) the DASH diet constituted the primary dietary intervention; (2) the study population comprised pregnant women; (3) data on gestational hypertension and pregnancy outcomes were reported; and (4) the study design was either randomized controlled trials (RCTs) or observational studies. Exclusion criteria encompassed duplicate data, unclear or incomplete information, animal studies, studies lacking a control group, and reviews without primary data. The study selection process is depicted in the PRISMA flow diagram (Figure 1).

### 2.4. Quality Assessment

Non-randomized studies were evaluated using the Newcastle–Ottawa Scale (NOS), which assesses participant selection, comparability of groups, and outcome or exposure ascertainment. Higher NOS scores indicated higher study quality. Results of the quality assessment are presented in Table 1 [19]. The risk of bias in RCTs was assessed using the Cochrane Risk of Bias Tool, which evaluates domains, including random sequence generation, allocation concealment, blinding, incomplete outcome data, selective reporting, and other bias sources. Each domain was rated as “Low,” “High,” or “Unclear” risk of bias per Cochrane Handbook guidelines. Detailed assessments are presented in Table 2 [20].

### 2.5. Data Extraction Methodology

For data analysis the control group was defined as the cohort with low adherence to the DASH diet (Q1) when available; otherwise comparisons were made with other dietary patterns. The intervention group consisted of participants with the highest DASH adherence (Q4 or Q5) or overall DASH compliance, when comparisons were made against other dietary strategies. When data were stratified by gestational age, the 8th-to-13th-week interval was selected. Where statistical models adjusted for confounders were used, models controlling for the greatest number of confounders were chosen. Data extraction included study author location, date, methodology, population characteristics, DASH adherence and outcomes comparing low versus high adherence or conventional diet.

### 2.6. Data Synthesis and Statistical Analysis

To calculate pooled relative risks (RR), study weights, and heterogeneity, data were initially organized into tables including RR, 95% confidence intervals (CI), and sample sizes (N). Data were log-transformed [log (RR)], and standard errors (SE) were computed using SE = (log (upper CI) − log (lower CI))/3.92. Study weights were calculated under a random-effects model using the DerSimonian–Laird method [21]. The fixed-effects model was deemed inappropriate given the presence of clinical and methodological heterogeneity, implying that true effect sizes vary among studies. The overall RR was computed as the weighted mean of log (RR) and then back-transformed (e^{mean}) to RR. Heterogeneity was assessed via the I^2^ statistic and Cochran’s Q-test. Statistical analyses and forest plots were generated using the ‘meta’ package in R version 4.4.3 with the functions metagen () and forest (). A funnel plot to test for publication bias was created in the R statistical computing environment, utilizing the specialized metafor package. The analysis strictly adhered to raw data to ensure unbiased, precise results [22].

### 2.7. Sensitivity and Subgroup Analysis

Subgroup analyses were conducted to explore the association between adherence to the DASH dietary pattern and specific hypertensive disorders of pregnancy, particularly preeclampsia. In addition, a sensitivity analysis was conducted to evaluate the robustness of the pooled estimates in relation to study design. This approach was used to determine whether the overall findings were influenced by the inclusion of heterogenous study designs.

## 3. Results

### 3.1. Study Selection

Figure 1 presents the flowchart of the study selection process and the reasons for article exclusion. Initially, 642 publications were identified. After removing duplicate studies, 361 publications remained. Titles and abstracts were then screened, and 72 articles were selected for further examination based on their full texts. Ultimately, 5 studies met all selection criteria and were included in the meta-analysis [23,24,25,26,27].

### 3.2. Study Quality Assessment

Table 1 summarizes the quality scores of each observational study according to the NOS.

**Figure 2:**
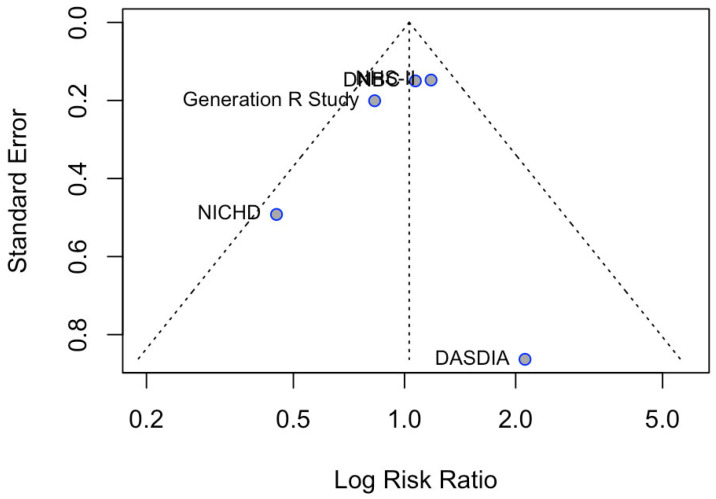
Funnel plot for publication bias.

Due to the small number of included studies (n = 5), formal statistical testing (Egger’s test) for publication bias was not performed. Although visual inspection of the funnel plot suggests obvious asymmetry, these results should be interpreted cautiously (Figure 2).

### 3.3. Study Characteristics

Table 3 and Table 4 for summarize the main characteristics and findings of the included studies. The four prospective cohort studies [23,24,25,26] and one randomized clinical trial [27] included in this review were conducted between 1991 and 2020, incorporated populations with diverse demographic and clinical profiles. Notably, variations were observed in participants’ age and in the presence of pre-existing metabolic disorders, such as obesity and diabetes mellitus. The follow-up duration ranged from a few years to two decades, allowing for the evaluation of both short-term and long-term effects of the DASH diet. Additionally, differences were observed in age distribution between the low- (Low DASH) and high-adherence (High DASH) groups, with women in the latter group generally exhibiting a higher mean age.

### 3.4. DASH Diet Assessment and Exposure Period

As presented in Table 5, the included studies employed diverse DASH scoring systems, dietary assessment instruments, and exposure periods. Four out of five studies [23,24,25,26] utilized food frequency questionnaires (FFQs), with one study additionally including the Automated Self-Administered 24 h Dietary Assessment Tool (ASA24) [24], while the DASDIA trial in Brazil employed 24 h dietary recalls combined with an adherence evaluation tool [27]. Scoring systems generally assigned 1–5 points per component (covering fruits, vegetables, low-fat dairy, red/processed meats, sugar-sweetened beverages, sodium, whole grains, and nuts/legumes) based on intake quintiles within the study population. In contrast, the DASDIA trial scored adherence from 0–4 points per visit, reflecting quantity, variety, meal patterns, and gestational weight gain, with higher scores indicating greater adherence [27]. The exposure periods varied, ranging from periconception and early pregnancy to mid-pregnancy or the entire duration of the intervention until delivery, highlighting heterogeneity in dietary assessment timing across studies.

### 3.5. Gestational Hypertension Incidence

**Figure 3:**
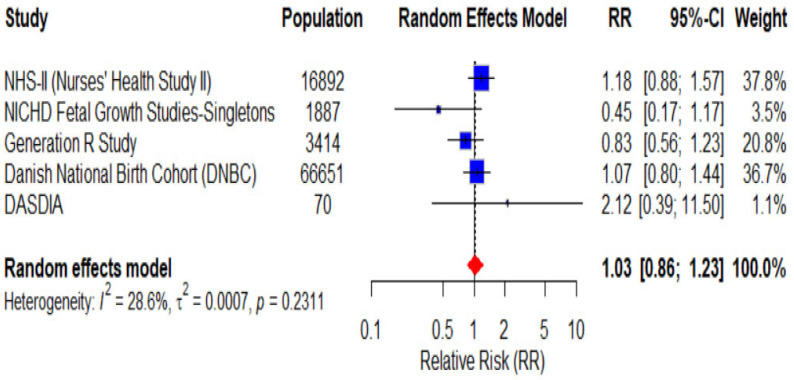
Forest plot presenting the pooled RR of the 5 included studies for a total of 88,914 pregnant women for gestational hypertension using a random-effects model.

This meta-analysis includes five studies with a total population of 88,914 participants, demonstrating notable variability in sample size and the statistical power of individual estimates [23,24,25,26,27]. The reported RRs range from 0.45 to 2.12, indicating substantial heterogeneity in the estimated effects. The overall pooled RR, based on the random-effects model, is 1.03 (95% CI: 0.86–1.23), suggesting no statistically significant association between exposure and outcome. Between-study heterogeneity is moderate (I^2^ = 28.6%) and statistically non-significant (*p* = 0.2311). The forest plot of the analysis for gestational hypertension is presented in Figure 3.

### 3.6. Subgroup Analysis Results for Preeclampsia

**Figure 4:**
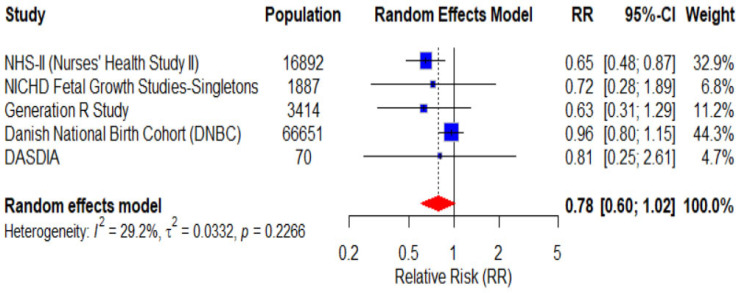
Forest plot presenting the pooled RR of the 5 included studies for a total of 88,914 pregnant women for preeclampsia.

The RRs range from 0.63 to 0.96 with a pooled RR of 1.03 (CI 0.60–1.02, *p* = 0.2266), indicating a potential protective association between adherence to the DASH diet and the occurrence of preeclampsia, although the analysis did not yield a statistically significant result. Intra-study heterogeneity is moderate (I^2^ = 29.2%) and not statistically significant (*p* = 0.227). The forest plot of the analysis for preeclampsia is presented in Figure 4.

### 3.7. Sensitivity Analysis Results for Observational Studies

**Figure 5:**
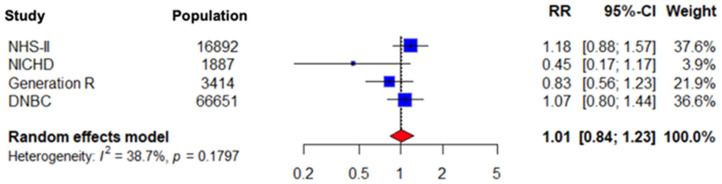
Forest plot presenting the pooled RR of the 4 included observational studies for gestational hypertension.

**Figure 6:**
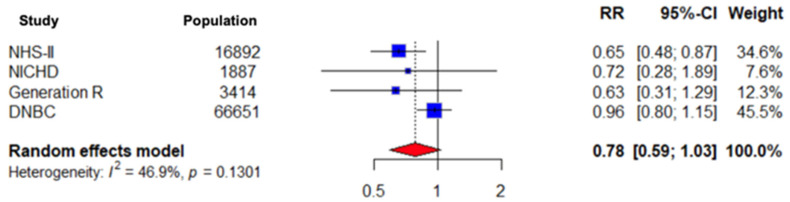
Forest plot presenting the pooled RR of the 4 included observational studies for preeclampsia.

Given the inclusion of a single randomized controlled trial alongside observational studies, a sensitivity analysis was performed excluding the RCT to assess the robustness of the pooled estimates. The sensitivity analysis including only observational studies yielded results consistent with the primary analysis, indicating that exclusion of the randomized controlled trial did not materially alter the overall findings both for gestational hypertension and pre-eclampsia (RR 1.01; CI 0.84–1.23, *p* = 0.1797 and RR 0.78; CI 0.59–1.03, *p* = 0.1301, respectively). The results are presented in Figure 5 and Figure 6 below.

## 4. Discussion

This meta-analysis of data from five included studies reveals an interesting, albeit statistically non-significant, trend regarding gestational hypertension. Although there is an observed trend toward a more positive outcome in the DASH diet group, the results must be interpreted cautiously. Similarly, in the case of preeclampsia, even though the results were also in favor of a possible protective effect of this nutritional regime, statistical significance was not achieved. These findings should be interpreted in the right context, as the observed associations are exploratory in nature and should not be overemphasized in terms of clinical importance in the absence of conclusive evidence.

Although, as already mentioned, the observational studies included in our meta-analysis did not demonstrate statistically significant associations between adherence to the DASH dietary pattern and the risk of gestational hypertension or preeclampsia, available data from interventional studies remain conflicting. Regarding blood pressure regulation, the randomized trial by Santos et al. suggests a potential protective effect of the DASH diet, particularly when combined with sodium restriction. Specifically, in the included RCT enrolling 60 pregnant women with gestational or chronic hypertension, adherence to the DASH diet for two months led to a significant reduction in blood pressure and preeclampsia incidence (26.7% vs. 53.3%) [27]. Similarly, in the study by Najafian et al. (2023), adherence to a sodium-restricted DASH diet (4 g/day) for 12 weeks in 85 women with gestational hypertension was associated with a significantly lower incidence of preeclampsia (43.2% vs. 65.9%) [28]. In contrast, the study by Jiang et al. (2019), which included 114 obese pregnant women without sodium restriction, found no difference in the incidence of gestational hypertension or preeclampsia between the intervention and control groups (8.9% vs. 8.6%, *p* = 0.95), and similar negative findings were reported by Vesco et al. (2014) in a comparable population (44% vs. 38%, *p* > 0.05) [29,30].

At the level of placental microcirculation, it has been suggested that the effect of this dietary pattern during pregnancy may be more detectable at the level of fetoplacental microvascular adaptation, rather than on gross blood pressure outcomes alone. Higher adherence to the DASH diet was associated with slightly lower umbilical artery pulsatility index in mid- and late pregnancy, although no meaningful associations were observed with uterine artery Doppler indices. Even though the fetoplacental vasculature is entirely regulated by the endothelium, uteroplacental perfusion is also influenced by autonomic factors, meaning that improvements in endothelial-dependent placental vascular tone may not immediately translate into clinically measurable reductions in maternal blood pressure, particularly in low-risk populations [25].

Regarding clinical hypertensive outcomes, as mentioned in the study by Fulay et al. (2018), no significant relationship between DASH diet adherence during the first trimester and the development of hypertensive disorders later in pregnancy was observed [31]. This lack of association may be due to the powerful physiological processes taking place during pregnancy, such as hormonal fluctuations (progesterone and estradiol) and alterations in the renin-angiotensin system (RAS), which could potentially mask the subtle or long-term protective effects of the dietary pattern [31]. Furthermore, the timing of dietary assessment remains a critical point; for example a dietary intervention may need to be initiated or sustained during the period of maximal blood pressure risk to be effective.

Interestingly, a protective effect against PE is observed in pregnant women with pre-existing cardiometabolic disorders (such as chronic hypertension or gestational diabetes). However, this benefit appears weaker or non-existent in healthy pregnancies [32]. Baseline cardiometabolic risk seems to play an important role in the response to DASH diet interventions during pregnancy and influence the overall risk for adverse events. Specifically, in a meta-analysis investigating the role of the DASH diet in glycemic control, participants with gestational diabetes, obesity, or hypertensive disorders exhibited distinct metabolic profiles at baseline, including elevated fasting plasma glucose, increased insulin resistance, and higher blood pressure. The DASH diet significantly reduced fasting plasma glucose and decreased the incidence of preeclampsia, macrosomia, and large-for-gestational-age infants, particularly in those with higher baseline metabolic risk. These effects may be mediated by the diet’s higher content of magnesium, calcium, and arginine, its low-glycemic composition, and its favorable fatty acid profile, which can improve insulin sensitivity and blood pressure regulation. Conversely, outcomes such as Homeostatic Model Assessment for Insulin Resistance (HOMA-IR), gestational age, and cesarean section rates were not significantly affected, suggesting that the magnitude of cardiometabolic derangement may influence which outcomes respond to dietary intervention [17].

The above tendency was also observed in the studies included in this review. Baseline cardiometabolic risk was a key modifier of the association between maternal DASH diet adherence and blood pressure during pregnancy. Li et al. reported that women with higher baseline BMI, lower education, and higher rates of smoking tended to have lower DASH scores and higher mid- and late-pregnancy blood pressure, while higher DASH adherence was associated with lower mid-pregnancy diastolic blood pressure [24]. Similarly, Wiertsema et al. found that women in the lowest DASH quartile had higher pre-pregnancy BMI, more overweight/obesity, and higher cardiometabolic risk factors, which corresponded to higher mid- and late-pregnancy blood pressure (after adjustment for these baseline factors); most associations were attenuated except for mid-pregnancy diastolic blood pressure [25].

Our study has limitations. Firstly, our research was able to retrieve only a small number of studies providing patient data on GH outcomes compared to DASH diet adherence. In the investigation for publication bias, while the funnel plot exhibited asymmetry, suggesting potential publication bias, this interpretation must be approached with caution given the limited number of studies, which may also reflect differences in study populations rather than missing data. Additionally, the majority of included studies were observational in design, which inherently limits the ability to establish causality and increases susceptibility to residual confounding. An important consideration in interpreting our findings is the combination of four observational cohort studies with a single small randomized controlled trial. While all included studies assessed DASH diet adherence and hypertensive disorders of pregnancy using comparable exposure definitions and outcomes, the heterogeneity in study design may influence the validity of pooled estimates. To address this, we conducted a sensitivity analysis excluding the RCT, which yielded results consistent with the primary analysis. Dietary exposures were also assessed using heterogeneous methods, and definitions of dietary patterns (including DASH) were not consistently applied across studies, reducing comparability. Importantly, the timing of dietary exposure varied across studies, and insufficient reporting made stratified or sensitivity analyses based on exposure timing not possible, despite the potential for differential physiological effects across gestational periods.

Future research should focus on monitoring dietary patterns earlier, ideally before conception, and rely on more objective assessment tools, such as biomarkers. This approach could provide a clearer understanding of whether, when, and how diet may exert a protective effect.

## 5. Conclusions

The available evidence does not support a statistically significant association between adherence to the DASH diet and reduced risk of gestational hypertension. Although some studies suggest potential benefits, the limited number of high-quality studies hinders definitive conclusions. Further randomized controlled trials and well-designed prospective studies are needed to thoroughly investigate the possible relationship between the DASH dietary pattern and gestational hypertension. Therefore, based on current evidence, the DASH diet cannot yet be specifically endorsed for the prevention of gestational hypertension, beyond general dietary recommendations for a healthy pregnancy. Despite the absence of statistically significant results, the present study highlights an important gap in the literature and underscores the need for continued research in this area.

## Figures and Tables

**Table 1:** Quality ratings of observational studies based on the NOS scale.

Study	Authors	Study Quality	NOS Score
NHS-II (Nurses’ Health Study II)	Arvizu et al.	Very good	9/9
NICHD Fetal Growth Studies—Singletons	Li et al.	Very good	9/9
Generation R Study	Wiertsema et al.	Very good	9/9
DNBC (Danish National Birth Cohort)	Arvizu et al.	Very good	9/9

**Table 2:** Risk of bias assessment using the Cochrane RoB2 tool for randomized controlled trials.

Study	Authors	Risk of Bias
DASDIA	Santos et al.	Unclear risk of bias

**Table 3:** Summary of characteristics and findings of the included studies.

First Author	Population, N	Study Design	Main Findings	Follow-Up	Confounding Factors	Conclusions
Arvizu et al.	11,535	Prospective cohort study	Reduced risk of GH and preeclampsia in highest DASH quintiles	1991–2009	Age, BMI, physical activity, energy intake, smoking, prior pregnancy	DASH diet associated with lower risk of preeclampsia, no significant effect on GH. Highest risk in low DASH adherence
Arvizu et al.	66,651	Prospective cohort study	No significant difference in GH risk across DASH categories	Jan 1996- Oct 2002	Age, pre-pregnancy BMI, smoking, diabetes status, etc.	No significant association between DASH and GH or preeclampsia risk
Li et al.	1887	Prospective cohort study	No significant association with GH; reduced preeclampsia risk with high DASH adherence	2009–2013	Maternal age, race, education, marital status, pre-pregnancy BMI, physical activity, sleep, energy intake	DASH diet reduces risk of preeclampsia, but not GH
Wiertsema et al.	3414	Prospective cohort study	Lower systolic and diastolic BP in Q4 DASH score; no significant difference in HDP	2002–2006	Maternal age, BMI, smoking, alcohol, education, folic acid, energy intake	DASH diet may benefit BP during pregnancy but has no significant effect on hypertensive disorders
Santos et al.	70	RCT	No significant differences in development of HDP of pregnancy between groups.	2016–2020	Diabetes type (1 vs. 2), Diabetes onset time, Pre-pregnancy BMI, History of preeclampsia, Socioeconomic status and ethnicity	DASH diet had no significant effect on preventing HDP in pregnant women with diabetes. Some genetic and phenotypic factors were linked to increased HDP risk

DASH, Dietary Approaches to Stop Hypertension; GH, Gestational Hypertension; BMI, Body Mass Index; vs., versus; RCT, Randomized Controlled Trial; HDP, Hypertensive Disorders of Pregnancy.

**Table 4:** Population Characteristics and Incidence of Gestational Hypertension in Relation to the DASH Diet.

First Author	Country	Gestational Week (Weeks)	Age (Years)	Low DASH (Age)	High DASH (Age)	High DASH, N	High DASH, N	GH Low DASH %	GH High DASH, %
Arvizu et al.	Denmark	25	30 ± 4	30 ± 4	31 ± 4	14.685	14.586	1%	0.9%
Arvizu et al.	USA	>20	34.6	34.1 (3.9)	35.1 (3.8)	2135	2333	3.3%	3.9%
Li et al.	USA	16–22	28.1	25.5 ± 5.43	30.4 ± 4.84	1682	404	6.2%	5.8%
Santos et al.	Brazil	<28	32 (25.7–36.0)	31 (25.0–35.0)	34 (28–37)	31	34	4.9%	10.3%
Wiertsema et al.	Netherlands	8–13	31.4 (4.4)	29.7 (5.0)	32.5 (3.8)	860	920	6.3%	5.2%

GH, Gestational Hypertension; DASH, Dietary Approaches to Stop Hypertension.

**Table 5:** DASH diet assessment and exposure period in the individual studies.

First Author	Study	DASH Assessment Instrument	Scoring System	Exposure Period
Arvizu et al.	DNBC	FFQ	Sum of 1–5 points per component (fruits/fruit juices, vegetables, low-fat dairy, red/processed meats, SSBs, sodium, whole grains, nuts/legumes) based on intake quintiles within the study population	The closest FFQ preceding each pregnancy
Arvizu et al.	NHS-II (Nurses’ Health Study II)	FFQ (at median 25 wks)	Sum of 1–5 points per component (fruits/fruit juices, vegetables, low-fat dairy, red/processed meats, SSBs, sodium, whole grains, nuts/legumes) based on intake quintiles within the study population	Previous 4 weeks from FFQ
Li et al.	NICHD Fetal Growth Studies—Singletons	FFQ (8–13 wks), ASA24 (16–22 and 24–29 wks)	Sum of 1–5 points per component (fruits/fruit juices, vegetables, low-fat dairy, red/processed meats, SSBs, sodium, whole grains, nuts/legumes) based on intake quintiles within the study population	8–13 wks, 16–22, 24–29 wks.
Santos et al.	DASDIA	24 h dietary recalls and adherence evaluation tool (4 items: quantity, variety, meals, gestational weight gain)	Score 0–4 points per visit based on adherence to DASH components (high adherence ≥2, low-to-moderate <2)	From inclusion (approx. <28 wks gestation) until delivery
Wiertsema et al.	Generation R	FFQ (at median 13 wks)	Sum of 1–5 points per component (fruits/fruit juices, vegetables, low-fat dairy, red/processed meats, SSBs, sodium, whole grains, nuts/legumes) based on intake quintiles within the study population	3 mo prior to enrollment (periconception and early pregnancy)

FFQ, Food Frequency Questionnaire, ASA24, Automated Self-Administered 24 h Dietary Assessment Tool; wks, weeks; mo, months.

## Data Availability

No new data were created or analyzed in this study. Data sharing is not applicable to this article.
